# Human African Trypanosomiasis Presenting at Least 29 Years after Infection—What Can This Teach Us about the Pathogenesis and Control of This Neglected Tropical Disease?

**DOI:** 10.1371/journal.pntd.0003349

**Published:** 2014-12-18

**Authors:** Darshan Sudarshi, Sarah Lawrence, William Owen Pickrell, Vinay Eligar, Richard Walters, Shumonta Quaderi, Alice Walker, Paul Capewell, Caroline Clucas, Angela Vincent, Francesco Checchi, Annette MacLeod, Michael Brown

**Affiliations:** 1 Hospital for Tropical Diseases, University College London Hospital, London, United Kingdom; 2 Morriston Hospital, Swansea, Wales, United Kingdom; 3 Princess of Wales Hospital, Bridgend Hospital, Wales, United Kingdom; 4 Institute of Biodiversity, Animal Health and Comparative Medicine, University of Glasgow, Glasgow, United Kingdom; 5 Nuffield Dept of Clinical Neurology, University of Oxford, Oxford, United Kingdom; 6 Faculty of Infectious & Tropical Diseases, London School of Hygiene & Tropical Medicine, London, United Kingdom; Emory University, United States of America

## Introduction

The World Health Organization (WHO) has recently announced its plan to eliminate Human African trypanosomiasis (HAT). The plan's main focus is on the *Trypanosoma brucei gambiense* subspecies, which causes 97% of cases [1]. However, total elimination from certain areas has proved extremely difficult previously; there has been ongoing parasite detection in several foci in West Africa, despite effective screening programmes. This has focussed interest on the potential role of asymptomatic human carriers in contributing to transmission. In this report, we present a unique case of a patient harbouring the parasite for more than three decades before developing stage 2 sleeping sickness [2].

## Presentation Of Case

**Figure 1:**
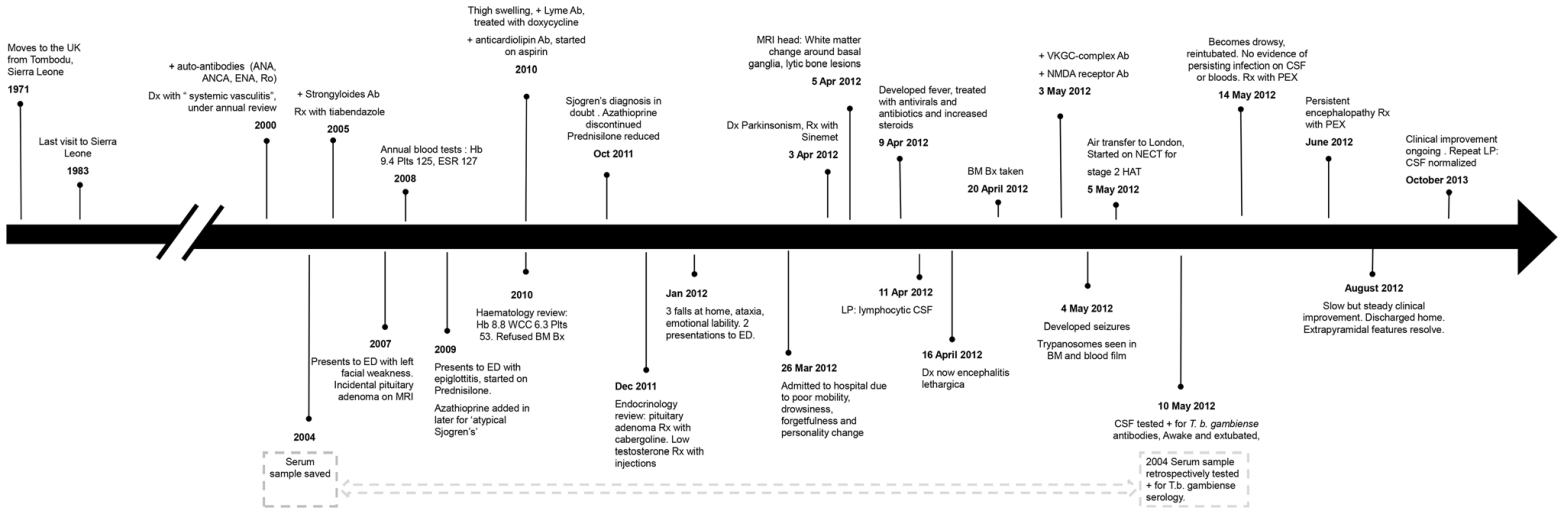
Timeline of the patient's clinical course. Timeline is not to scale. Abbreviations: Ab: Antibody ANA: anti-nuclear antibody, ANCA: anti-neutrophil cytoplasmic antibody, BM Bx: bone marrow biopsy, CSF: cerebrospinal fluid, ED: Emergency department, ENA: extractable nuclear antigens, ESR: erythrocyte sedimentation rate, Hb: haemoglobin, LP: lumbar puncture, MRI: magnetic resonance imaging, NECT: nifurtimox-eflornithine combination therapy, NMDA: N-methyl-D-aspartate receptor, PEX: Plasma exchange, PCR: polymerase chain reaction, Plts: platelets, Rx: treated with, VGKC: complex voltage gated potassium channel-complex, WCC: white cell count.

A 62-year-old male (see Fig. 1 for summary timeline) was admitted to Bridgend hospital in Wales in March 2012. He presented with a 3-month history of worsening mobility, shuffling gait, fatigue, somnolence, emotional lability, and personality change. Born in Eastern Sierra Leone, he had moved to the United Kingdom in 1971, making two visits back to his home country, the last of which was for 3 months in 1983, with no subsequent travel to Africa.

In 2000, during investigation of venous leg ulcers, he was found to have a normocytic anaemia (Hb 10.4 g/dl, (13.0–17.0)), and erythrocyte sedimentation rate (ESR) of 78 mm/hour, (1–20). Elevated titres of anti-nuclear antibodies (1∶100), anti-nuclear cytoplasmic antibodies (1∶160), and Anti-Ro antibodies were detected. There were no sicca symptoms and no specific treatment for autoimmune disease was instituted. He was kept under annual review; the only medication given was tiabendazole in 2005 for a persistent eosinophilia and positive *Strongyloides* serology.

In 2007 a pituitary adenoma (with a normal hormone profile) was detected on magnetic resonance imaging (MRI) of the brain during investigation of a unilateral lower motor neurone seventh nerve palsy. In 2008 a diagnosis of “atypical Sjögren's syndrome” was made in light of persistent autoantibodies. At this stage his haemoglobin was 9.4 g/dl, platelets 125×10^9^/L (150–400), ESR 127 mm/hr, Immunoglobulin M (IgM) levels were 20.6 g/L (0.4–2.3), and Immunoglobulin G (IgG) levels 32.6 g/L (7.0–16.0). In 2009 he was started on prednisolone 10 mg daily following an episode of epiglottitis. Azathioprine was added and increased to a dose of 100 mg daily.

In 2010 the patient developed unilateral thigh swelling and a transient rash. *Borrelia burgdorferi* serology (IgM & IgG) was positive and he received 2 weeks of doxycycline. Elevated anticardiolipin antibodies were detected and he was prescribed aspirin (subsequently discontinued due to epistaxis). A bone marrow examination was advised to investigate persistent anaemia and thrombocytopenia, but the patient declined.

In 2011 osteoporosis was discovered on a Dual-energy X-ray absorptiometry (DEXA) scan; low testosterone levels and high prolactin levels (70,400 U/L) prompted treatment with testosterone gel and cabergoline. MRI of the brain showed no change in the pituitary adenoma. The “Sjögren's” diagnosis was reconsidered, azathioprine was discontinued and prednisolone reduced to 2.5 mg daily. Until this point in the history, the patient had remained relatively well, with no symptoms beyond those mentioned above. Indeed, his wife stated “he had never missed a day of work in his life.”

In January 2012, the patient developed unsteadiness and had three falls at home. He was admitted to hospital in March 2012 with rapidly deteriorating mobility, alteration in his sleep–wake cycle, and episodes of drowsiness. Neurological examination demonstrated rigidity, bradykinesia, and a right lateral gaze palsy. An initial diagnosis of Parkinsonism resulted in treatment with carbidopa/levodopa with minimal benefit. A few days into his admission, he developed fevers and longer sustained episodes of unresponsiveness. He was treated for possible meningoencepalitis with aciclovir and ceftriaxone. Hydrocortisone (400 mg daily) was commenced to cover pituitary apoplexy which was subsequently excluded on imaging. A cerebrospinal fluid examination revealed 250 lymphocytes/mm^3^, protein 0.57 g/L, and a normal glucose ratio; gram stain, bacterial culture, and PCR for herpes viruses were negative.

An MRI of the brainrevealed nonspecific white matter changes around the basal ganglia and multiple radiolucent lesions in the skull vault. Serum and urine electrophoresis did not detect a paraprotein, and a skeletal survey was normal. HIV and treponemal serologies were negative. Since the patient continued to experience symptoms of drowsiness, a diagnosis of encephalitis lethargica was entertained. Further results demonstrated high levels of voltage gated potassium channel–complex (VGKC-complex), antibodies, and moderate levels of N-methyl-D-aspartate (NMDA) receptor antibodies. He developed further episodes of unresponsiveness, and a generalised tonic-clonic seizure followed by persistent coma prompted transfer to a neurology ward with a view to intravenous immunoglobulin for a presumed autoimmune encephalitis. Meanwhile, microscopy of a bone marrow trephine demonstrated multiple *Trypanosoma brucei* trypomastigotes, subsequently seen in the peripheral blood. He was intubated, and on 5 May, urgent airborne transfer to the critical care unit at the Hospital for Tropical Diseases in London was arranged.

Treatment was commenced immediately with NECT (nifurtimox-eflornithine combination therapy), with oral nifurtimox 5 mg/kg every 8 hours and intravenous eflornithine 200 mg/kg every 12 hours, which is the recommended first-line therapy for stage 2 HAT [1]. NECT was chosen over the eflornithine monotherapy (the alternative treatment for stage 2 HAT), since it has shown to be equally efficacious whilst causing less severe haemotological side effects. Furthermore, treatment with NECT is of shorter duration and requires fewer infusions, minimizing the chance of line related complications [1].

On Day 4 of the Intensive Therapy Unit (ITU) admission, the patient was promptly extubated and transferred to the ward shortly afterwards, with complete reversal of coma and resolution of pancytopenia. Cerebrospinal fluid (CSF) examination revealed a positive indirect fluorescent antibody test (IFAT) for *T. b. gambiense*, with a titre of 1/32, and serum IFAT was positive at 1/3200. Analysis of a stored serum sample from 2004 demonstrated a *T. b. gambiense* IFAT titre of 1/800.

On Day 9 of NECT treatment the patient started to become drowsy once again, requiring reintubation. A computed tomography (CT) scan of the brain showed no acute changes. Repeat CSF and blood exams showed no evidence of trypanosomes. Nonconvulsive status was excluded by electroencephalogram. Because of the laboratory evidence of effective treatment and the overlap in clinical features of autoimmune encephalitis with persistently high VGKC-complex antibodies, plasma exchange (PEX) was commenced. After four cycles of PEX, the patient showed clinical improvement and was extubated. Unfortunately, following further neurological deterioration, the patient was reintubated and a surgical tracheostomy inserted. Repeat lumbar puncture showed improvement of CSF markers (protein 0.43 g/L, white cells 8/mm^3^) with no trypanosomes observed in CSF or blood. All subsequent films were negative. Sleep–wake cycle disturbance continued for the next few weeks with occasional apnoeic episodes, and resulted in a decision to administer a further five cycles of PEX, which was followed by sustained recovery (associated with a decline in VGKC-complex antibodies below the “positive” threshold of 100pM) and discharge home in August 2012. At outpatient review 1 month later, all extrapyramidal features had resolved, with considerable improvement in mobility and cognition. Repeat CSF examinations in February (CSF: Protein 0.27, 8 lymphocytes, CSF *T. b. gambiense* IFAT 1∶8) and October 2013 (CSF: protein 0.27, 0 lymphocytes, CSF *T.b.gambiense* IFAT 1∶8) reflected the ongoing clinical recovery.

## Case Discussion

### Trypanotolerance

To date, this case represents the longest duration of HAT infection ever reported. As our patient last visited an endemic country in 1983 and became unwell in 2012, the period from infection to stage 2 symptoms must have been at least 29 years.

**Figure 2:**
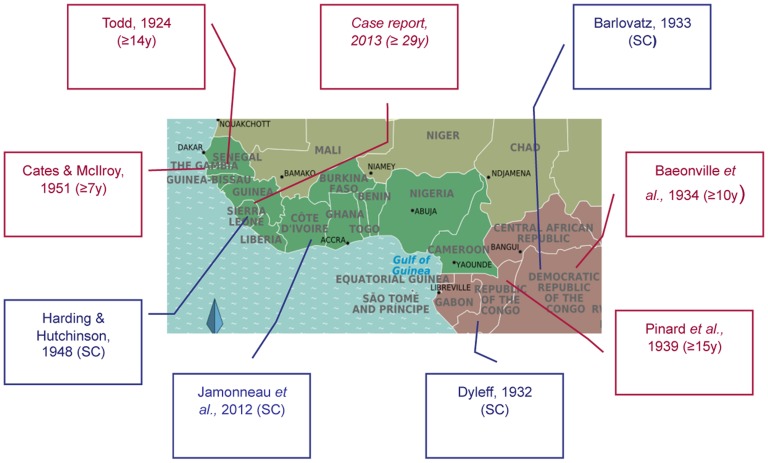
Map showing reports of spontaneous clearance of infection (blue squares), or extended duration of infection (red squares). Abbreviations: sc: spontaneous clearance of infection, y: years. References: [4]–[11]. Adapted from [3].

**Figure 3:**
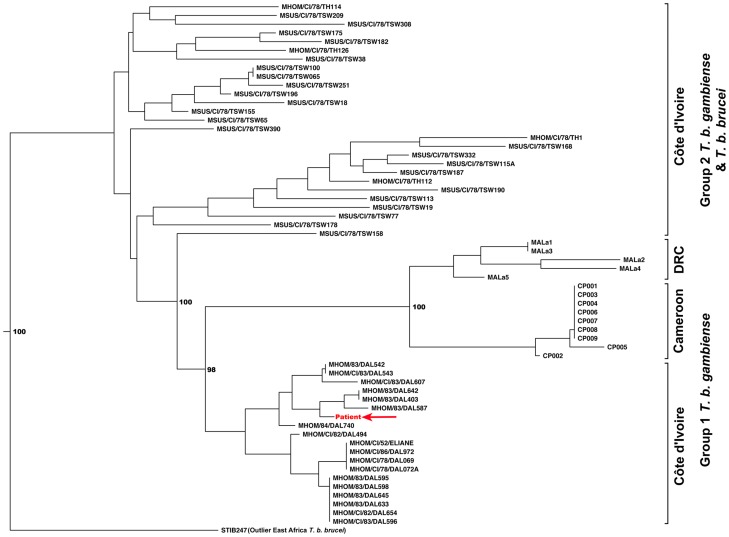
Phylogenetic tree depicting the relationship between our patient's parasite and field isolates taken between 1978–1983. Neighbour joining tree of *T. b. brucei*, *T. b. gambiense* group 1 and *T. b. gambiense* group 2 isolates from Cote d'Ivoire and *T. b. gambiense* group 1 isolates from Cameroon and the Democratic Republic of Congo. Our patient's isolate clustered most closely with other Type 1 isolates from Cote d'Ivoire.

This case provides concrete evidence behind the emerging concept of “human trypanotolerance,” which challenges the traditionally held view that, left untreated, HAT always progresses to the fatal stage 2 phase. Checchi, et al., reviewed the prior trypanotolerance literature, which consists of cases showing an extended duration of infection, like the one discussed above, as well as cohorts demonstrating spontaneous resolution of infection [3]. We have summarised and updated the key evidence in this review, illustrated geographically in Fig. 2 [4]–[12]. In our case, we were able to demonstrate the presence of long standing HAT infection, using several molecular techniques that were not available in the past. First, historically, it had not been possible to confirm whether chronic infections were due to pathogenic trypanosome species, as diagnosis was based on microscopy, which does not allow differentiation of sub-species [3]. However, PCR amplification of our patient's parasite DNA clearly indicated *T. b. gambiense* group 1 [13]. Second, retrospective testing of a serum sample in 2004 demonstrated clear evidence of serological infection prior to disease. Third, additional microsatellite typing showed clustering with field isolates taken from West Africa in the 1980s (Fig. 3), which is the estimated time our patient would have been first infected. Our findings are consistent with a recent 15-year follow-up study of untreated stage 1 HAT patients in the Ivory Coast, which showed that whilst some patients progress to stage 2, a proportion of patients become asymptomatic carriers, and another subset appears to self cure [7].

Elucidating the genetic basis underlying trypanotolerance is of great interest. Although few parasite factors have been discovered, associations between human genes involved in the immune response and susceptibility to HAT have been reported [14],[15].

### Implications For Public Health And Disease Control

Current disease control strategies rely on case finding and treatment of patients with detectable parasites in lymph nodes or blood or laboratory evidence of stage 2 disease [16]. Such strategies could fail to stop transmission if there exist hidden populations of individuals that harbour undetectably low parasite numbers and act as reservoirs of infection [2],[17]. Evidence that seropositive, microscopy-negative patients have low-level infection is supported by molecular diagnostic work demonstrating detectable circulating *T. b. gambiense* DNA in a proportion of these asymptomatic carriers [7],[18]. Their potential role in disease transmission is supported by a parasite population genetic study [19] as well as by research in trypanotolerant cattle [20]. Detecting asymptomatic carriers is particularly challenging due to the lack of a specific diagnostic test that can be used in remote field settings. However, a recent study using blood stored on filter paper with the immune trypanolysis test has shown promising results [21].

As the focus of HAT programs shifts towards elimination of the disease, it is essential that trypanotolerance is taken into account [22]. Specifically, the following questions will need to be answered: How common are these asymptomatic carriers? Does their prevalence vary in different HAT foci? How can they best be detected and monitored? What is the attributable fraction of transmission associated with failure to detect and treat such cases?

## Immunopathology

Several features of this case generate hypotheses about the immunopathology of HAT.

The factors that led to our patient's development of stage 2 disease after such a long duration of chronic infection are unknown, but it is feasible that immunosuppressive therapy (azathioprine and prednisolone) may have played a role. While immunosuppressive therapy is well described when used for other protozoal infections [23], this is the first such case reported for HAT. This may be due to the low prevalence of HAT in populations at risk of iatrogenic immunosuppression. HIV and HAT have considerable geographic overlap; limited evidence does not suggest an increased risk of HAT among HIV+ persons [24], but there is no data on the role of HIV in activation of latent HAT infections, a mechanism well established for Chagas trypanosomiasis [25]. One can speculate that the immunosuppressive therapy somehow altered the tight balance between immune control and disease.

The complex clinical course of our patient before diagnosis of HAT warrants attention. For more than a decade, our patient was diagnosed with a series of conditions (Sjögren's, Lyme, antiphospholipid disease) based on abnormal serological tests. We consider these to be false-positive results, due to nonspecific immune activation characteristic of long-standing HAT infection. Through continuous antigenic variation of their variant surface glycoprotein, trypanosomes evade the immune system, inducing polyclonal B cell activation [26], which produces hypergammaglobulinaemia and autoantibodies [27],[28].

We hypothesise that the clinical course of our patient after definitive treatment may be similarly related to an autoimmune component. Most patients presenting with stage 2 HAT in field settings respond to treatment with rapid improvement within days. Whereas it is not uncommon for patients given melarsoprol to develop a post-treatment reactive encephalopathy, such a syndrome has been described as less frequent with NECT [29]. Our patient had an uncharacteristically slow improvement following NECT with a fluctuating sleep disorder lasting several weeks. We believe that this could be due to immune-mediated encephalopathy. Indeed, our patient was found to be positive for VGKC-complex antibodies, which are known to cause autoimmune encephalitis [30]. This led us to administer plasma exchange, with clinical improvement mirroring the fall in antibody titres. One might postulate that VGKC-complex antibodies may therefore contribute to stage 2 immunopathogenesis. However, a stored serum sample from 5 years before onset of stage 2 symptoms was also strongly positive (1628 pM; normal range <100 pM), so other insults must be required to generate pathology, such as the blood-brain barrier breakdown that must have occurred during the trypanosomal invasion. Improved understanding of CNS immunopathology is important, as it may help to design better and safer treatments.

## Conclusion

Although we report only on a single patient, this case adds to the accumulating evidence of human trypanotolerance, thus further stimulating debate about the impact of this phenomenon in the field and illuminating new avenues of research that must be explored if we are to provide the answers that are vital for the elimination of this neglected tropical disease.

**Key Learning Points**

- This case represents the longest duration of *T.b gambiense* infection ever reported
- It adds strong evidence behind human trypanotolerance, which has important implications for disease elimination programmes
- This unique case has revealed interesting aspects of the immunopathology of HAT, which necessitate further research
